# Rare Thrombotic Complications of COVID-19: A Case Series

**DOI:** 10.7759/cureus.22637

**Published:** 2022-02-26

**Authors:** Sruthi Vellanki, Anup Kumar Trikannad Ashwini Kumar, Ryan Stoffel, Sravya Vellanki, Zachary Nuffer

**Affiliations:** 1 Internal Medicine, Union Hospital, Terre Haute, USA; 2 Medicine, Indiana University School of Medicine, Terre Haute, USA; 3 Pulmonary and Critical Care, Johns Hopkins University, Baltimore, USA; 4 Radiology, Union Hospital, Terre Haute, USA

**Keywords:** blue toe syndrome, bowel ischemia, thromboembolic complications, sars-cov-2, covid-19

## Abstract

Coronavirus disease 2019 (COVID-19) is known to manifest with bilateral pneumonia and acute respiratory distress syndrome. This infection with severe acute respiratory syndrome coronavirus 2 (SAR-CoV-2) is alarming because it not only affects the respiratory system but may also cause thromboembolic events. Multiple studies have reported procoagulation/hypercoagulable complications in COVID-19. This case series is a valuable addition to the literature because it reflects unique presentations of thrombotic events in COVID-19 patients. We report two cases in which patients presented with thromboembolic complications secondary to COVID-19 infection: one with severe bowel ischemia and the other with blue toe syndrome. To formulate management strategies to prevent fatal outcomes for patients with COVID-19, physicians must be vigilant in identifying life-threatening thromboembolic complications from this disease.

## Introduction

Severe acute respiratory syndrome coronavirus 2 (SARS-CoV-2) causes an aggressive infection with potentially serious health consequences, coronavirus disease 2019 (COVID-19). Although COVID-19 primarily causes respiratory infection, recent studies have shifted their attention to vascular complications of this disease, including alarming thromboembolism [1]. This case series underscores the importance of recognizing hypercoagulability as a cause of arterial thrombus presenting as bowel ischemia and blue toe syndrome in patients with COVID-19. This report highlights the need for early diagnosis of vascular complications and prompt management to avoid poor outcomes.

## Case presentation

Case 1

A 66-year-old man presented to the hospital with a two-day history of abdominal pain, nausea, vomiting, cough, and shortness of breath. He was tested positive for COVID-19 before hospitalization. Physical examination was normal except for diminished breath sounds on auscultation; his oxygen saturation was 98% on room air. A therapeutic dose of enoxaparin was initiated for an elevated D-dimer (3.12 mg/L).

Computed tomography (CT) of the abdomen and pelvis on presentation demonstrated no abnormal gastrointestinal findings. Chest CT angiography (CTA) was obtained to evaluate for pulmonary embolism in the setting of an elevated D-dimer. Chest CTA was negative for acute pulmonary embolism but visualized patchy bilateral ground-glass consolidation with a peripheral predominance, compatible with COVID-19 pneumonia.

**Figure 1:**
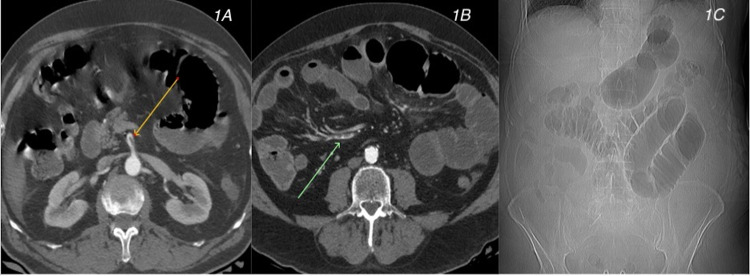
Case 1. CT angiography shows intraluminal thrombus within the celiac trunk (yellow arrow in 1A) and ileocolic artery (green arrow in 1B). CT (scout view) (1C) shows focal small bowel ileus compatible with ischemia. CT, computed tomography

On hospital day 2, the patient reported worsening abdominal pain associated with nausea and vomiting. His D-dimer continued to be elevated and was increased to 7.24 mg/L. Repeat CT of the abdomen and pelvis with intravenous contrast showed acute intraluminal thrombus involving the superior mesenteric artery (SMA) and left colic artery in the setting of abnormally dilated loops of small bowel with air-fluid levels, compatible with ischemic ileus (Figure 1). The patient was emergently transferred by air evacuation to a tertiary care center for vascular surgery evaluation.

On arrival, the patient was evaluated for SMA thrombectomy and stent placement. A hyperemic loop of the small bowel was identified in the left lower quadrant, and a bowel perforation was discovered. There was clear evidence of full-thickness small bowel ischemia, with evidence of extensive transmural necrosis. He underwent SMA mechanical thrombectomy followed by damage control laparotomy and small bowel resection. On postoperative day 2, he underwent reopening of laparotomy and was found to have patchy ischemia throughout most of the small bowel as well as a segment of ischemic bowel with perforation. At that time, his abdomen was washed out, small bowel resection was performed, and two small bowel anastomoses were created with the placement of ABTHERA™ negative-pressure wound therapy. On postoperative day 4 following the initial procedure, staged relaparotomy was performed. During abdomen exploration, distal small bowel anastomosis breakdown was identified and the bowel was resected. The patient was taken to the operating room for restoration of the abdominal continuity and abdominal closure. The patient’s postoperative course was complicated by new-onset atrial fibrillation with a rapid ventricular rate, in addition to deterioration of the anastomoses with stool leakage. The family declined further surgical intervention and approved transfer to hospice care, and the patient died on day 10.

Case 2

A 62-year-old man with a history of chronic obstructive pulmonary disease, tobacco use, chronic respiratory failure, and Parkinson's disease presented to the hospital with progressive weakness, dyspnea, and cough. He tested positive for COVID-19. Physical examination was normal except for diminished breath sounds on auscultation. Chest CTA in the setting of elevated D-dimer (4.4 mg/L) was negative for acute pulmonary embolism but demonstrated patchy bilateral ground-glass consolidation, compatible with COVID-19 pneumonia. He was started on dexamethasone, a therapeutic dose of enoxaparin, and remdesivir.

**Figure 2:**
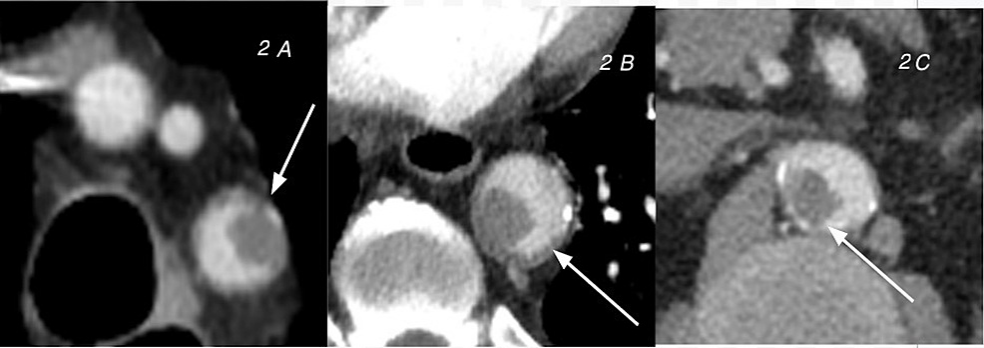
Case 2. CT angiography per the pulmonary embolism protocol shows free-floating thrombosis in the arch (A), descending aorta (B), and abdominal aorta (C). CT, Computed tomography

The patient had tachypnea with a respiratory rate of 34 breaths per minute and was using accessory muscles of respiration. He was transferred to the critical care unit where he was intubated and placed on mechanical ventilatory support. His D-dimer continued to be elevated and increased to 9.2 mg/L. Repeat chest CTA demonstrated new findings of many intraluminal aortic thrombi (Figure 2). These findings raised concern for embolic ischemic disease.

The patient continued to receive a therapeutic dose of enoxaparin in addition to bronchodilator treatments and intravenous steroids. After a lengthy stay of 25 days in the hospital, the patient signed himself out against medical advice. Anticoagulation was continued; however, the patient was nonadherent to anticoagulation. He later returned to the emergency room with pain, swelling, and dark discoloration of his right fifth toe. Because this presentation was concerning for dry gangrene, the patient opted for a partial amputation of his fifth right toe at the distal interphalangeal joint. The surgery was successful, and the patient was discharged home; in addition, his COVID-19 related respiratory symptoms had resolved. The patient made a good postoperative recovery and was seen in the clinic in two weeks with good surgical outcomes.

## Discussion

Infection with SARS-CoV-2 induces proinflammatory cytokines and/or procoagulant factors that can activate the coagulation cascade, leading to thrombosis, atherosclerotic plaque rupture, and ischemia, and COVID-19 can lead to severe illness with accompanying multiorgan failure. Inflammatory cytokine release mediates atherosclerosis and results in hemodynamic changes because of the induction of procoagulant mediators. It is believed that a storm of inflammatory mediators such as cytokines triggered by SARS-CoV-2 results in irreversible multisystem inflammation that can partially present as abnormal hemostasis and coagulopathy, further aggravating organ injury [1].

As a viral infection, COVID-19 induces a complete neurovascular disease syndrome that initiates a viral attack on the angiotensin-converting enzyme 2 receptors of the microvascular system, resulting in two distinct processes: (1) microvascular thrombosis and inflammation of the endothelium and (2) microvascular thrombosis in the lungs, which causes ventilation/perfusion mismatch and results in hypoxemia. The inflammatory component further enhances the thrombotic process in all organs. Imaging CT in combination with assessment of D-dimer levels provides biomarkers to diagnose and monitor these prothrombotic and embolic processes of COVID-19 [2].

In case 1, results of the abdominal and pelvic CTA at presentation were normal despite the patient having abdominal symptoms. His D-dimer level continued to rise with ongoing abdominal symptoms, which prompted us to repeat imaging, yielding a diagnosis of acute SMA thrombosis. As a life-threatening condition that may result from atherosclerotic or embolic disease, the mortality rate in SMA thrombosis ranges from 28% to 90% [3,4]. The classic clinical finding is severe abdominal pain that is disproportionate to physical examination findings. The diagnosis is most often confirmed with CTA. In case 1, the patient developed embolic SMA disease in the setting of COVID-19. Treatment includes anticoagulation and emergent surgical evaluation. Current evidence supports rapid endovascular revascularization to quickly reestablish blood flow, followed by surgical exploration with resection of necrotic bowel, if present, as was performed in our patient [5,6].

In case 2, the chest CTA at presentation did not show emboli. Repeat chest CTA was performed in the setting of an elevated D-dimer, which showed aortic thrombi. The patient later presented with blue toe syndrome, which is characterized by tissue ischemia secondary to atherothrombotic or cholesterol crystal embolization. These embolic/thrombotic events lead to the occlusion of small vessels, limiting distal arterial perfusion and thus causing tissue ischemia [7,8]. The causes of the embolic/thrombotic events that lead to blue toe syndrome can be categorized as emboli from the cardiac and arterial system, acquired hypercoagulability disorders, and syndromes that lead to peripheral vascular pathology [8]. In our case, the patient acquired atherothrombotic blue toe syndrome most likely in the setting of COVID-19 infection.

The optimal medical management of blue toe syndrome consists of anticoagulant therapy with either low-dose subcutaneous heparin or combination therapy with aspirin and dipyridamole antiplatelet therapy. However, with embolic causes, optimal management of blue toe syndrome is still uncertain. Some clinician-researchers recommend early surgery to remove the embolic source to spare the risk of further tissue or limb loss. Other clinicians suggest that surgical management is often unnecessary, and medical management with antiplatelet therapy is satisfactory in a subset of patients as well [9]. Current evidence supports prompt anticoagulation therapy for the management of blue toe syndrome, followed by close observation to determine if the patient needs expedited surgical management for worsening tissue ischemia or requires amputation for gangrenous lesions [7]. In our patient, amputation was deemed necessary because of progression to the gangrenous lesion, which was worsened by his nonadherence to anticoagulation therapy.

Based on the literature review, there are a great number of studies that addressed the association between COVID-19 and bowel ischemia. Acute mesenteric ischemia does seem to be a rare complication of COVID-19, and elevated D-dimer levels can be considered while assessing a patient of COVID-19 with abdominal pain for mesenteric ischemia [10]. However, more prospective studies are required to assess the association between elevated D-dimer in the setting of COVID-19 and bowel ischemia. Further, there are limited review articles to associate elevated D-dimer levels in the setting of COVID-19 and limb ischemia.

## Conclusions

As noted in both of our cases, it is crucial to repeat imaging based on elevated D-dimer levels in COVID-19 patients. Further studies are needed to define the specific correlation between markers of coagulopathy such as D-dimer in patients with COVID-19 and outcomes after thrombotic events. Available evidence indicates that a dysfunctional coagulation pathway in COVID-19 patients results in embolic events; however, specific studies concerning vascular complications are lacking. It is crucial to have large multicenter studies to investigate vascular complications in COVID-19 and to formulate management strategies with good patient outcomes.
